# A Clinical Prediction Model of Overall Survival for Patients with Cervical Cancer Aged 25–69 Years

**DOI:** 10.3390/medicina59030600

**Published:** 2023-03-17

**Authors:** Wenli Fan, Qin Lu, Guokun Liu

**Affiliations:** 1 Wuhan Children’s Hospital (Wuhan Maternal and Child Healthcare Hospital), Tongji Medical College, Huazhong University of Science and Technology, Wuhan 430000, China; 2 Ultrasound Room, Huai’an Second People’s Hospital, The Affiliated Huai’an Hospital of Xuzhou Medical University, Huai’an 223002, China; 3 Comprehensive Ward, Huai’an Second People’s Hospital, The Affiliated Huai’an Hospital of Xuzhou Medical University, Huai’an 223002, China

**Keywords:** cervical cancer, disease prediction, machine learning, predict overall survival

## Abstract

*Aims:* This study aims to develop a prediction tool for the overall survival of cervical cancer patients. *Methods:* We obtained 4116 female patients diagnosed with cervical cancer aged 25–69 during 2008–2019 from the Surveillance, Epidemiology, and End Results Program. The overall survival between groups was illustrated by the Kaplan–Meier method and compared by a log-rank test adjusted by the Bonferroni–Holm method. We first performed the multivariate Cox regression analysis to evaluate the predictive values of the variables. A prediction model was created using cox regression based on the training set, and the model was presented as a nomogram. The proposed nomogram was designed to predict the 1-year, 3-year, and 5-year overall survival of patients with cervical cancer. Besides the c-index, time-dependent receiver operating curves, and calibration curves were created to evaluate the accuracy of the nomogram at the timepoint of one year, three years, and five years. *Results:* With a median follow-up of 54 (28, 92) months, 1045 (25.39%) patients were deceased. Compared with alive individuals, the deceased were significantly older and the primary site was more likely to be the cervix uteri site, large tumor size, higher grade, and higher combined summary stage (all *p* values < 0.001). In the multivariate Cox regression, age at diagnosis, race, tumor size, grade, combined summary stage, pathology, and surgery treatment were significantly associated with the all-cause mortality for patients with cervical cancer. The proposed nomogram showed good performance with a C-index of 0.82 in the training set. The 1-year, 3-year, and 5-year areas under the curves (with 95% confidence interval) of the receiver operating curves were 0.88 (0.84, 0.91), 0.84 (0.81, 0.87), and 0.83 (0.80, 0.86), respectively. *Conclusions:* This study develops a prediction nomogram model for the overall survival of cervical cancer patients with a good performance. Further studies are required to validate the prediction model further.

## 1. Introduction

Cervical cancer is the fourth most common malignant tumor in women, leading to a substantial health threat worldwide [1,2]. Owing to the advances in prevention, diagnosis, and treatment, the incidence and mortality of cervical cancer have decreased by at least half in the past three decades in developed countries [1]. Still, the disease remained a significant health burden on a global scale. It was reported that 569,847 patients were newly diagnosed with cervical cancer, and 311,365 deaths were caused by cervical cancer worldwide in 2018.

Squamous cell carcinoma accounts for the major histological subtypes (about 70%), and adenocarcinoma is the second most common subtype accounting for about 25% [1,3]. Many factors have been previously reported to be associated with the survival of this malignancy, such as lymph node metastasis, histologic type, tumor size, etc. [4]. However, the overall survival varies among cervical cancer patients at the individual level, even for those with the same disease stage and histologic type. A single predictive biomarker alone is insufficient to evaluate the disease’s survival comprehensively.

As a class of artificial intelligence, machine learning uses algorithmic methods to make machines perform disease prediction without programming [5]. Applying machine learning to big data provides a powerful method for evaluating complex healthcare information [6]. Therefore, this study aims to develop a prediction tool for the overall survival of cervical cancer patients based on machine learning.

## 2. Methods

### 2.1. Data Source

The Surveillance, Epidemiology, and End Results (SEER) Program collects population-based cancer incidence and survival from the US cancer registries, which cover about 48% of the total US population. Patient demographics, tumor site, morphology, and stage at diagnosis, treatment, and follow-up survival status were routinely collected in the SEER registries. This study obtained data from the “Incidence—SEER Research Data, 8 Registries, Nov 2021 Sub (1975–2019)”. The cervical cancer diagnosis was based on the International Classification of Diseases for Oncology, 3rd Edition (ICD-O-3). We included participants who were (1) pathologically diagnosed with cervical cancer, (2) aged between 25–69, (3) with complete survival records, and (4) newly diagnosed between 2008–2019. Exclusion criteria were (1) diagnosed only by autopsy or death certificate, (2) without race, tumor site, size, grade, or stage record, and (3) missing surgery record. In the SEER database, the surgery records of participants were recorded as (1) Yes (have received surgery treatment), (2) No (have not received surgery treatment), or (3) Not available (have no information about surgery treatment). We excluded patients without available information on surgery. It should be noted that “missing surgery record” indicated that we are not sure if the participant received surgery treatment, instead of that they have not received surgery. Follow-up time was defined as the time from diagnosis to death or the last contact date. Finally, 4116 female patients diagnosed with cervical cancer during 2008–2019 were included in this study.

In this study, sociodemographic, pathologic, and clinical variables were obtained for further analysis, including age at diagnosis, race (White, Black, and other races), primary site (cervix uteri, endocervix, exocervix, and overlapping lesion), tumor size, grade (Grade I, well-differentiated; Grade II, moderately differentiated; Grade III, poorly differentiated; Grade IV, undifferentiated), combined summary stage (regional, localized, and distant), pathology (squamous cell carcinoma, adenocarcinoma, and others), and surgical treatment. All the data were acquired from the SEER database by SEER*Stat software (version 2.4.0).

### 2.2. Development and Validation of the Prediction Model

We first performed the multivariate Cox proportional hazard regression model to evaluate the predictive values of the variables. Multiple biomarkers were input to the Cox regression model, including age at diagnosis, race, primary site, tumor size, grade, combined summary stage, pathology, and surgical treatment. The results were shown by hazard ratios (HRs) with 95% confidence intervals (CIs). Multicollinearity refers to the high correlation between two or more predictor variables in a regression model. Multicollinearity can lead to unstable estimates of the regression coefficients, which makes it difficult to determine the true effect of each predictor variable on the outcome variable. The variance inflation factor is a measure widely used to assess the degree of multicollinearity in a regression model. We calculated the variance inflation factors of each variable to evaluate the multicollinearity. The variance inflation factor value of 1 indicates no correlation, between 1 and 5 indicates moderation correlation, above 5 indicates high correlation.

The input data were randomly divided into a training set and a testing set at a 7:3 ratio. The training set (*n* = 2882) was used to create the prediction model, while the testing set (*n* = 1234) was used to validate the model performance. The prediction model was created by cox regression and was presented as a nomogram. The proposed nomogram was designed to predict the 1-year, 3-year, and 5-year overall survival of patients with cervical cancer. Besides the c-index, time-dependent receiver operating, and calibration curves were created to evaluate the accuracy of the nomogram at the timepoint of one year, three years, and five years.

### 2.3. Statistical Analysis

Descriptive statistics were used to describe the baseline characteristics. We represented the continuous variables as mean ± standard deviation and categorical variables as percentages. The baseline characteristics were compared by the Kruskal–Wallis test or chi-square test as appropriate. The overall survival between the group was illustrated by the Kaplan–Meier method and compared by a log-rank test adjusted by the Bonferroni–Holm method. We used the Bootstrapping 1000 resamples to validate the performance of the proposed model internally. *p* values < 0.05 were considered statistical significance. All statistical analyses were performed in R software (version 4.0).

## 3. Results

### 3.1. Participant Characteristics

With a median follow-up of 54 (28, 92) months, 1045 (25.39%) patients were deceased. Compared with the alive individuals, the dead were significantly older and more likely to be in the cervix uteri site, large tumor size, higher grade, and higher combined summary stage (all *p* values < 0.001). The baseline participant characteristics are shown in Table 1.

### 3.2. Cox Regression Analysis

**Figure 1:**
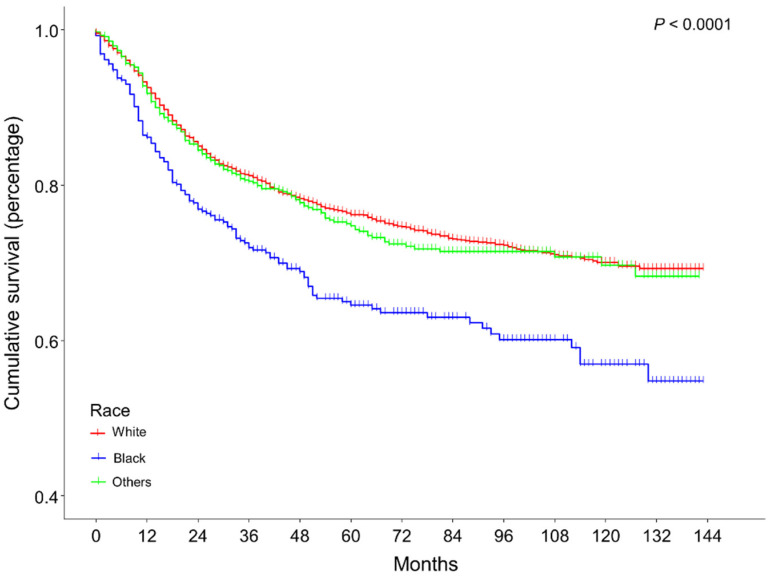
K m plotter of overall survival for patients with cervical cancer in different races. The overall survival for cervical cancer patients of different races (log-rank *p* value < 0.0001). The survival was significantly lower in the black race than in the white (BH-adjusted *p* value < 0.001) and other races (BH-adjusted *p* value < 0.001). However, no statistical difference was observed between the white race and other races (BH-adjusted *p* value = 0.62).

In the multivariate Cox regression, age at diagnosis, race, tumor size, grade, combined summary stage, pathology, and surgery treatment were significantly associated with the all-cause mortality for patients with cervical cancer. The results of the multivariate Cox regression analysis are shown in Table 2. Compared with the white race, black race patients were at a 1.37 (1.14–1.65)-fold risk of all-cause death. Furthermore, Figure 1 illustrates the overall survival of cervical cancer patients of different races (log-rank *p* value < 0.0001). The survival was significantly lower in the black race than in the white (BH-adjusted *p* value < 0.001) and other races (BH-adjusted *p* value < 0.001). However, no statistical difference was observed between the white race and other races (BH-adjusted *p* value = 0.62). Additionally, the variance inflation factors of each variable were provided in the Supplementary Table S1. Moderately differentiated and poorly differentiated grade showed high multicollinearity with variance inflation factor values of 5.4 and 5.8, respectively. Therefore, we input all variables into the prediction model.

### 3.3. Development and Validation of the Prediction Model

**Figure 2:**
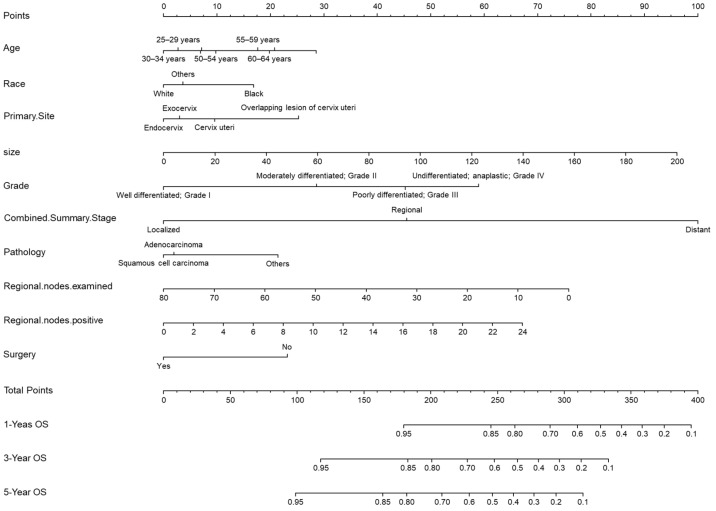
A nomogram for predicting the overall survival probability in patients with cervical cancer.

A prediction model was created by cox regression based on the training set, and the proposed model was presented as a nomogram in Figure 2. Multiple variables were input: age at diagnosis, race, primary site, tumor size, grade, combined summary stage, pathology, and surgical treatment. The C-index in the training set was 0.82.

**Figure 3:**
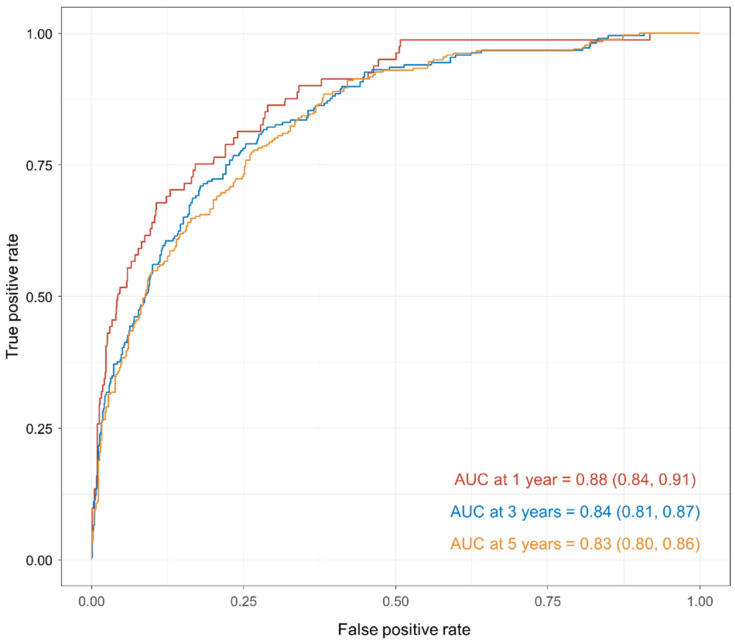
Time-dependent receiver operating curves on the 1-year, 3-year, and 5-year model performance in the validation set.

**Figure 4:**
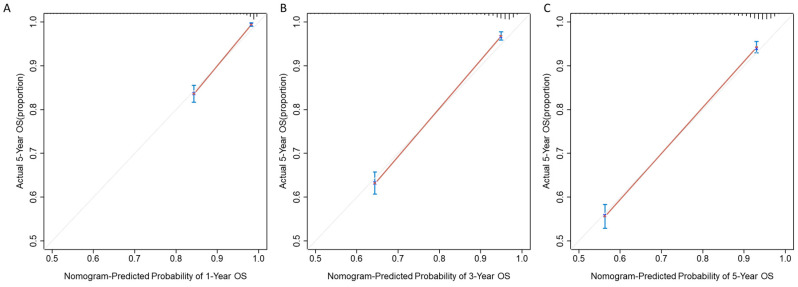
Calibration curve of the nomogram model at the timepoint of (**A**) 1 year, (**B**) 3 years, and (**C**) 5 years.

In the testing set, the nomogram showed good performance with a C-index of 0.81. Figure 3 shows that the performance remains satisfied at the time point of one year, three years, and five years, with the area under the curves (AUCs) of 0.88 (0.84, 0.91), 0.84 (0.81, 0.87), and 0.83 (0.80, 0.86). Additionally, we showed the calibration plots of the nomogram in Figure 4. The sensitivity and specificity of the model in the testing set are shown in Table 3. Our results showed that the nomogram had good calibration when predicting 1-year, 3-year, and 5-year overall survival probability.

## 4. Discussion

In this study, we obtained 4116 female patients diagnosed with cervical cancer during 2008–2019 from the SEER Program. Based on cox regression analysis, we developed a prediction model and presented it as a nomogram. In the validation, the model showed good performance with a C-index of 0.82. The ROCs show that the performance remains satisfied at one year, three years, and five years, with AUCs of 0.89, 0.86, and 0.84.

Previous studies have revealed many risk factors to predict overall survival for patients with cervical cancer (e.g., lymph node metastasis, histologic type, tumor size) [4]. However, cervical cancer patients show distinct prognoses even for those with the same histologic type. Prediction using a single biomarker alone is insufficient to comprehensively evaluate the disease survival. Nomograms based on machine learning integrate multiple biomarkers to comprehensively evaluate disease prognosis [7,8,9]. The visualized method is designed to generate the precise prediction tailored to an individual patient, providing a simple-to-use tool for clinicians to predict overall survival [10]. Recently, many nomograms have been developed for cancer diagnosis and prognosis, which showed better performance than the traditional clinical stage system [11,12,13].

Few studies proposed prediction tools to evaluate the overall survival of cervical cancer [14,15]. Polterauer et al. [14] developed a nomogram to predict overall survival in cervical cancer patients diagnosed using 528 consecutive patients. Gynecologists and Obstetricians stage, tumor size, age at diagnosis, histologic subtype, lymph node ratio, and parametrial involvement were input to the prediction model as nomogram covariates. This model was internally validated using 1000 bootstrap resampling, and the c-index for overall survival was 0.72 (25th and 75th percentiles, 0.70 and 0.74) [14]. In another study by Kidd and colleagues [15], 234 cervical cancer patients were included to develop the nomograms. The proposed nomograms showed reliable performance for recurrence-free survival, disease-specific survival, and overall survival with C-indexes of 0.741, 0.739, and 0.658, respectively [15]. Compared with previous studies, we included a large population-based sample size, which made our results more reliable and might be applied to the general population. Importantly, our nomogram showed good performance with a C-index of 0.82 and 1-year, 3-year, and 5-year, AUCs of 0.89, 0.86, and 0.84, respectively. The proposed nomogram was convenient and can be easily converted into an online prediction tool, which would help clinicians to make treatment decisions.

Tumor stage and histology subtype are well-demonstrated risk factors for worse survival of cervical cancer patients. However, it remains uncertain whether older age reduces the overall survival [16,17,18]. The median age of cervical cancer diagnosis is 49 years, and cervical cancer is mostly diagnosed in patients aged between 35 to 44 years. Current guidelines recommend cervical cancer screening for women below 65 but not above 65 years [19]. Still, many patients were diagnosed at elder age (above 65 years), which accounts for about 20% of all patients [20]. Therefore, research is required to investigate the risk factors to predict the survival of cervical cancer. In a previous study on 43,350 cervical cancer patients, Quinn et al. [21] reported that increased age (particularly > 70 years) was associated significantly with decreased survival trends. The trend remains consistent when stratified by various tumor stages and histology subtypes [21]. In our study, patients aged 65–69 were at a 1.6-fold risk of all-cause death compared with 25–29 years.

Despite the advantages, some limitations should be noticed. First, this study is based on the SEER program, which is performed in the US. It remains unclear whether the model can be applied to other races. Second, the predictive factors were input in this model were all records from the SEER. However, this database did not collect some important predictive biomarkers for cervical cancer (especially the recently proposed ones). Third, the prediction model was validated in the internal validation set. The further validation based on an external dataset would be necessary. Last but not least, this study excluded participants with missing records, which might induce additional selection bias. Further studies are required to further validate the prediction model.

## 5. Conclusions

In the present study, we developed a prediction nomogram model for the overall survival of cervical cancer patients with a good performance. Further studies are required to validate the prediction model further.

## Figures and Tables

**Table 1:** Patients baseline characteristics.

	All Patients	Alive	Dead	*p* Value
N	4116	3071	1045	
Age				<0.001
25–29 years	220 (5.3%)	180 (5.9%)	40 (3.8%)	
30–34 years	461 (11.2%)	397 (12.9%)	64 (6.1%)	
35–39 years	562 (13.7%)	466 (15.2%)	96 (9.2%)	
40–44 years	657 (16.0%)	523 (17.0%)	134 (12.8%)	
45–49 years	576 (14.0%)	428 (13.9%)	148 (14.2%)	
50–54 years	547 (13.3%)	377 (12.3%)	170 (16.3%)	
55–59 years	434 (10.5%)	290 (9.4%)	144 (13.8%)	
60–64 years	366 (8.9%)	234 (7.6%)	132 (12.6%)	
65–69 years	293 (7.1%)	176 (5.7%)	117 (11.2%)	
Race				<0.001
White	3056 (74.2%)	2314 (75.4%)	742 (71.0%)	
Black	387 (9.4%)	251 (8.2%)	136 (13.0%)	
Others	673 (16.4%)	506 (16.5%)	167 (16.0%)	
Primary site:				<0.001
Cervix uteri	3031 (73.6%)	2179 (71.0%)	852 (81.5%)	
Endocervix	926 (22.5%)	769 (25.0%)	157 (15.0%)	
Exocervix	98 (2.4%)	81 (2.6%)	17 (1.6%)	
Overlapping lesion	61 (1.5%)	42 (1.4%)	19 (1.8%)	
Tumor size	30.0 (12.0, 54.2)	22.0 (9.0, 43.5)	54.0 (37.0, 71.0)	<0.001
Grade				<0.001
Grade I	658 (16.0%)	604 (19.7%)	54 (5.2%)	
Grade II	1920 (46.6%)	1518 (49.4%)	402 (38.5%)	
Grade III	1418 (34.5%)	886 (28.9%)	532 (50.9%)	
Grade IV	120 (2.9%)	63 (2.1%)	57 (5.5%)	
Combined summary stage				<0.001
Regional	1422 (34.5%)	906 (29.5%)	516 (49.4%)	
Localized	2257 (54.8%)	2054 (66.9%)	203 (19.4%)	
Distant	437 (10.6%)	111 (3.6%)	326 (31.2%)	
Pathology				<0.001
Squamous cell carcinoma	2564 (62.3%)	1874 (61.0%)	690 (66.0%)	
Adenocarcinoma	952 (23.1%)	807 (26.3%)	145 (13.9%)	
Others	600 (14.6%)	390 (12.7%)	210 (20.1%)	
Surgical treatment				<0.001
No	1257 (30.5%)	633 (20.6%)	624 (59.7%)	
Yes	2859 (69.5%)	2438 (79.4%)	421 (40.3%)	

**Table 2:** Cox regression on the predictive factors for survival of patients with cervical cancer.

	HR (95%CI)	*p* Value
Age		
25–29 years	Reference	
30–34 years	0.96 [0.64, 1.42]	0.823
35–39 years	1.08 [0.74, 1.56]	0.687
40–44 years	1.10 [0.77, 1.57]	0.599
45–49 years	1.39 [0.98, 1.98]	0.065
50–54 years	1.14 [0.81, 1.62]	0.448
55–59 years	1.31 [0.92, 1.87]	0.133
60–64 years	1.37 [0.96, 1.96]	0.083
65–69 years	1.59 [1.11, 2.28]	0.012
Race		
White	Reference	
Black	1.37 [1.14, 1.65]	0.001
Others	1.07 [0.90, 1.27]	0.434
Primary site		<0.001
Cervix uteri	Reference	
Endocervix	0.84 [0.69, 1.01]	0.069
Exocervix	0.89 [0.55, 1.44]	0.629
Overlapping lesion	1.27 [0.80, 2.01]	0.308
Tumor size	1.01 [1.01, 1.01]	<0.001
Grade		
Grade I	Reference	
Grade II	1.66 [1.24, 2.22]	0.001
Grade III	2.21 [1.65, 2.95]	<0.001
Grade IV	2.83 [1.92, 4.16]	<0.001
Combined summary stage		
Regional	Reference	
Localized	0.43 [0.36, 0.52]	<0.001
Distant	2.69 [2.32, 3.13]	<0.001
Pathology		
Squamous cell carcinoma	Reference	
Adenocarcinoma	1.03 [0.84, 1.26]	0.813
Others	1.46 [1.22, 1.73]	<0.001
Regional nodes positive	1.02 [0.99, 1.06]	0.226
Surgical treatment		<0.001
No	Reference	
Yes	0.57 [0.49, 0.67]	<0.001

HR, hazard ratios; CI, confidence interval.

**Table 3:** Model performance in the testing set.

	AUC (95%CI)	Sensitivity	Specificity
1 year	0.88 (0.84, 0.91)	0.85	0.71
3 years	0.84 (0.81, 0.87)	0.71	0.76
5 years	0.83 (0.80, 0.86)	0.47	0.78

AUC: area under the curves; CI: confidence intervals.

## Data Availability

The data used to support the findings of this study are acquired from the Surveillance, Epidemiology, and End Results Program.

## Supplementary Materials

The following supporting information can be downloaded at: https://www.mdpi.com/article/10.3390/medicina59030600/s1, Table S1: The variance inflation factors of each variable.
