# Supplementary material for: A Clinical Prediction Model of Overall Survival for Patients with Cervical Cancer Aged 25–69 Years
Source: Medicina (Kaunas). 2023 Mar 17;59(3):600. doi: 10.3390/medicina59030600 (PMC10052094; doi:10.3390/medicina59030600)
Supplement: Supplementary file 1 [file medicina-59-00600-s001.zip › medicina-2115289-supplementary.pdf]

Table S1. The variance inflation factors of each variable.

**Variable**	**Variance inflation factor**
Age=30-34 years	2.5
Age=35-39 years	3.1
Age=40-44 years	3.8
Age=45-49 years	4.1
Age=50-54 years	4.5
Age=55-59 years	4.0
Age=60-64 years	3.8
Age=65-69 years	3.5
Race=Black	1.1
Race=Others	1.1
Primary.Site=Endocervix	1.3
Primary.Site=Exocervix	1.0
Primary.Site=Overlapping lesion of cervix uteri	1.0
size	1.6
Grade=Moderately differentiated; Grade II	5.4
Grade=Poorly differentiated; Grade III	5.8
Grade=Undifferentiated; anaplastic; Grade IV	2.1
Combined.Summary.Stage=Localized	1.6
Combined.Summary.Stage=Distant	1.3
Pathology=Adenocarcinoma	1.4
Pathology=Others	1.3
Regional.nodes.examined	1.7
Regional.nodes.positive	1.3
Surgery=Yes	1.8
